# The Fluorescent Patient: An Unusual Effect of Fluorescein Angiography

**DOI:** 10.7759/cureus.15011

**Published:** 2021-05-13

**Authors:** Raphael Bertani, Carlos E Ferrarez, Caio M Perret, Sávio Batista, Stefan W Koester, Renan Maximillian Lovato, Marcelo Magaldi Ribeiro de Oliveira

**Affiliations:** 1 Neurosurgery, Hospital Municipal Miguel Couto, Rio de Janeiro, BRA; 2 Neurosurgery, Hospital Madre Teresa, Belo Horizonte, BRA; 3 Neurosciences, Federal University of Rio de Janeiro, Rio de Janeiro, BRA; 4 Neurosurgery, Vanderbilt University School of Medicine, Nashville, USA; 5 Neurosurgery, Santa Casa de São Paulo School of Medical Sciences, São Paulo, BRA

**Keywords:** fluorescein angiogram, fluorescein, fluorescein angiography, vascular neurosurgery, cerebrovascular surgery, open cerebrovascular neurosurgery, cerebrovascular diseases, moyamoya angiopathy, moyamoya, sta-mca bypass

## Abstract

Although fluorescein is widely used for intraoperative angiography, some of its side effects remain obscure. In this report, we present the case of a 41-year-old patient with chronic ischemia caused by moyamoya syndrome who underwent bypass revascularization with intraoperative fluorescein angiography (FA). Immediately after the surgery, the patient presented homogeneous fluorescence of the entire skin. We discuss this curious phenomenon as well as other side effects that may arise due to FA.

## Introduction

Fluorescein angiography (FA) is widely used in vascular and oncological neurosurgery [1,2,3], as well as ophthalmology [4]. It represents a more cost-effective solution when compared to indocyanine green (ICG), especially in low and middle-income settings. Based on the duration, outcome, and need for medical assistance, complications and side effects related to FA can be categorized into mild, moderate, or severe [5]. We describe a case in which a patient became entirely fluorescent after its use for video angiography during a superficial temporal artery to middle cerebral artery bypass surgery for moyamoya syndrome, a chronic vaso-occlusive crisis disease of unknown etiology that had caused chronic, diffuse cerebral ischemia in the patient. Although FA is a widely used substance for intraoperative angiography, some of its peculiar side effects may be unknown to most.

## Case presentation

**Figure 1:**
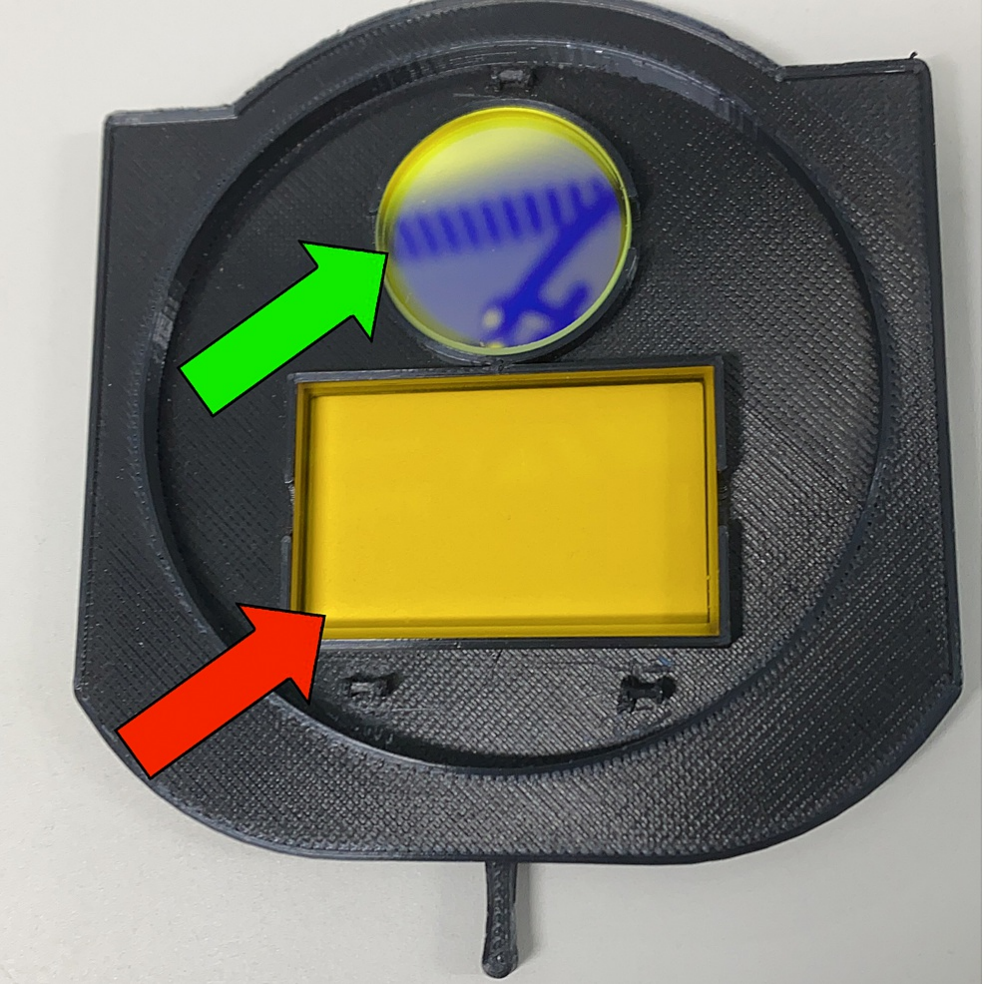
Custom-made filter Photograph showing the custom-made snap-on filter consisting of a yellow barrier filter (red arrow) and an ultraviolet filter (green arrow)

**Figure 2:**
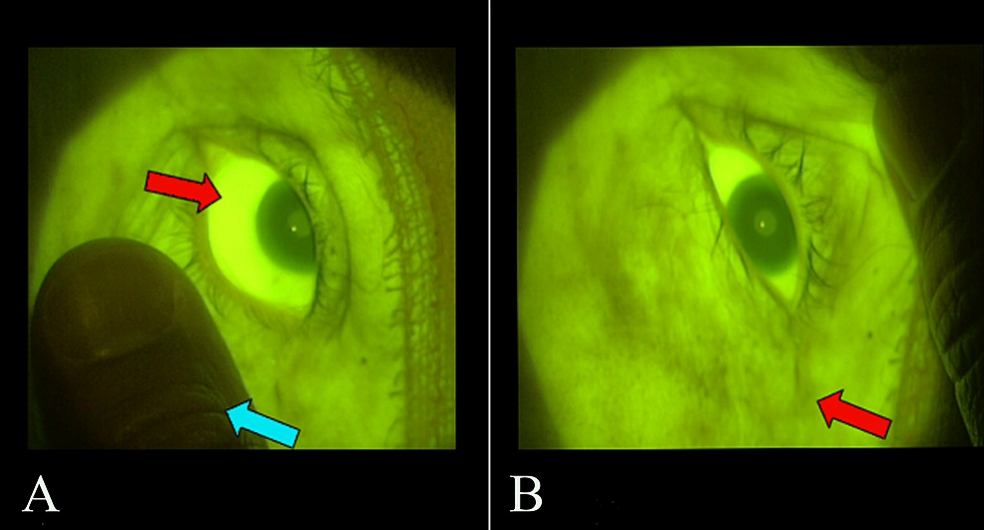
Fluorescence of patient's skin A photograph taken through the microscope and filter shows (A) fluorescence of the patient’s cornea (red arrow) as compared to examiner’s finger (blue arrow) and (B) fluorescence of the patient’s skin (red arrow)

A 41-year-old female patient with chronic ischemia caused by moyamoya syndrome was submitted to revascularization with a superficial temporal artery to middle cerebral artery bypass. With an adapted filter consisting of an ultraviolet filter and a yellow barrier filter (Figure 1) [3,6], FA was made possible with an S88® (Carl Zeiss Meditec, Jena, Germany) surgical microscope. Intravenous fluorescein was injected in a single 100-mg dose, allowing for the confirmation of bypass patency [7]. After the surgery, the patient’s skin and sclera were found to have acquired a yellowish hue (pseudojaundice). Examination through the microscope with the attached filter revealed fluorescence of the patient’s entire skin (Figure 2). No other symptoms were observed.

## Discussion

Even though FA is a widely used technique worldwide, especially in ophthalmology, there is a paucity of reports in the literature regarding benign skin complications that may arise from it. Kurli et al. [8] reported a case of skin discoloration and hypothesized that there could be a correlation between a previously diagnosed lymphoproliferative disorder and patchy skin discoloration. Saleh et al. [9] have reported a similar case in a patient who had not been diagnosed with other non-ophthalmologic pathologies, thus failing to link its occurrence to illnesses. Although most reported reactions seem mild, consisting of nausea, vomiting, mild allergic reactions, and accidental arterial injections, severe reactions with fluorescein have been previously reported in a survey from 1986 [10]. These included acute pulmonary edema, bronchospasms, anaphylaxis, myocardial infarction, seizures, and death [10]. A critical point with respect to this study is that death was considered to be related to fluorescein if the symptoms related to the cause of death occurred in the first 24-48 hours after the injection. Only one death was reported among the 222,000 patients included in the survey, and the exact cause was not disclosed. These nefarious complications have not been reported in the most recent studies, and until now, only 11 deaths have been documented in the medical literature pertaining to this subject [5,11].

Several mechanisms have been proposed to explain the adverse effects of fluorescein, including vasovagal reaction, drug allergy, histamine release, anxiety-related medullary sympathetic discharge, direct vasospastic toxic effect, and drug contamination [5]. However, none of them seem to fully explain the pseudojaundice phenomenon, for which further studies are needed in the field. Pseudojaundice has also been previously reported in the elderly and suggested as a potential differential diagnosis for pathological causes of jaundice [12,13]. Physicians can see a reaction similar to that of our patient in cases of accidental arterial fluorescein injections [14]. In such cases, fluorescein tends to stain the territory of irrigation belonging to that specific artery, associated with intense pain and skin discoloration [14]. In our case, the patient remained asymptomatic. The dye impregnated her skin diffusely, in a presentation consistent with pseudojaundice, other than patchy skin discoloration, associated with the fluorescence of the entire skin when observed with proper filter and lighting that lasted for several hours. To the best of our knowledge, there are no other reports of this unusual condition in the literature. Table 1 provides details about the fatalities related to fluorescein reported in the literature, and Table 2 summarizes complications associated with fluorescein as presented in the literature.

**Table 1:** Fluorescein-related deaths reported in the literature

Study	Cause of death	Comments
Ascaso et al. [15]	Myocardial infarction	The authors report the patient complained of chest pain 30 seconds after injection and collapsed due to cardiopulmonary arrest. Postmortem evaluation revealed a right coronary artery obstruction
Cummingham et al. [16]	Unclear	The patient suddenly collapsed due to cardiopulmonary arrest 20 minutes after an injection consisting of 5 mL of 10% fluorescein dye was administered. The patient had a history of stable angina and a previous myocardial infarction. Unfortunately, the authors could not obtain authorization for postmortem examination
Fineschi et al. [17]	Fatal anaphylactic shock	No history of previous allergies. Postmortem examination was consistent with anaphylaxis
Hitosugi et al. [18]	Fatal anaphylactic shock	The patient was pronounced dead two hours following fluorescein injection. Postmortem examination was consistent with anaphylaxis
Stein et al. [19]	Myocardial infarction	Death was reported several hours after the procedure; the relation to fluorescein injection remains unclear

**Table 2:** Complications correlated with fluorescein as described in the literature IgE: immunoglobulin E

Type of complication	Symptoms	Mechanism	Comments	References
Mild complications				
Mild allergic reaction	Nausea, vomiting, congestion, urticaria, wheezing, edema	Histamine release due to hypersensitivity	The incidence of adverse reactions in those with a history of allergic diseases seems to be higher	Nausea, vomiting, urticaria, localized reactions [3]; allergy [17,18]; hypersensitivity [19]; all [20]
Dermatological complications	Skin discoloration, phototoxic reaction, pseudojaundice	Arterial injection, production of free radicals upon exposure to certain wavelengths, unexplained mechanisms	Pseudojaundice mechanism remains unexplained	Pseudojaundice [4-8]; skin discoloration [5-7]; phototoxic reaction [21]
Severe reactions				
Severe allergic reaction	Anaphylaxis, bronchospasms	Several proposed mechanisms: vasovagal reaction, drug allergy, anxiety-related medullary sympathetic discharge, direct vasospastic toxic effect, drug contamination	IgE-mediated hypersensitivity	Hypersensitivity, same as above [17]; hypersensitivity, same as above [19]; all [20]
Severe non-allergic reaction	Myocardial infarction, seizures			Hypersensitivity, same as above [21]; severe reactions [13]

## Conclusions

We reported a case of a patient who presented with pseudojaundice and fluorescence of the skin and cornea following intraoperative FA; it lasted for several hours, but no treatment was required. FA has been used to a lesser extent in neurosurgery than ophthalmology, and gaining knowledge of its possible side effects is crucial for better communication with patients and managing complications. It seems to be a safe and cost-effective method of intraoperative video angiography. The differences between benign side effects and potentially harmful occurrences should be well understood and explained to the patients. New and updated survey studies should be performed to update current knowledge on this topic, especially on a global scale.

## References

[1] Narducci A, Onken J, Czabanka M, Hecht N, Vajkoczy P (2018). Fluorescein videoangiography during extracranial-to-intracranial bypass surgery: preliminary results. Acta Neurochir (Wien).

[2] Shah KJ, Cohen-Gadol AA (2019). The application of FLOW 800 ICG videoangiography color maps for neurovascular surgery and intraoperative decision making. World Neurosurg.

[3] Dijkstra BM, Jeltema HJR, Kruijff S, Groen RJM (2019). The application of fluorescence techniques in meningioma surgery-a review. Neurosurg Rev.

[4] Patz A, Finkelstein D, Fine SL, Murphy RP (1986). The role of fluorescein angiography in national collaborative studies. Ophthalmology.

[5] Kornblau IS, El-Annan JF (2019). Adverse reactions to fluorescein angiography: a comprehensive review of the literature. Surv Ophthalmol.

[6] Wolfe DR (1986). Fluorescein angiography basic science and engineering. Ophthalmology.

[7] Lovato RM, Araujo JL, Paiva AL, Veiga JC (2017). TMOD-24. Fluorescein guided surgery for malignant brain tumors: a case series with a low cost device. Neuro Oncol.

[8] Kurli M, Hollingworth K, Kumar V, Sandramouli S (2003). Fluorescein angiography and patchy skin discoloration: a case report. Eye (Lond).

[9] Saleh TA, Chidgey C, Wong KK (2004). Fluorescein angiography and patchy skin discoloration: a case report. Eye (Lond).

[10] Yannuzzi LA, Rohrer KT, Tindel LJ, Sobel RS, Costanza MA, Shields W, Zang E (1986). Fluorescein angiography complication survey. Ophthalmology.

[11] Kwan AS, Barry C, McAllister IL, Constable I (2006). Fluorescein angiography and adverse drug reactions revisited: the Lions Eye experience. Clin Exp Ophthalmol.

[12] Degelau J, Spilane M (1991). Geriatric pseudojaundice. J Am Geriatr Soc.

[13] Kalkan A, Turedi S, Aydin I (2015). Fluorescein-related extensive jaundice. Am J Emerg Med.

[14] Bovino JA, Marcus DF (1984). Accidental intra-arterial injection of fluorescein dye. Ophthalmic Surg.

[15] Ascaso FJ, Tiestos MT, Navales J, Iturbe F, Palomar A, Ayala JI (1993). Fatal acute myocardial infarction after intravenous fluorescein angiography. Retina.

[16] Cummingham EE, Balu V (1979). Cardiac arrest following fluorescein angiography. JAMA.

[17] Fineschi V, Monasterolo G, Rosi R, Turillazzi E (1999). Fatal anaphylactic shock during a fluorescein angiography. Forensic Sci Int.

[18] Hitosugi M, Omura K, Yokoyama T, Kawato H, Motozawa Y, Nagai T, Tokudome S (2004). An autopsy case of fatal anaphylactic shock following fluorescein angiography: a case report. Med Sci Law.

[19] Stein MR, Parker CW (1971). Reactions following intravenous fluorescein. Am J Ophthalmol.

[20] Meira J, Marques ML, Falcão-Reis F, Rebelo Gomes E, Carneiro Â (2020). Immediate reactions to fluorescein and indocyanine green in retinal angiography: review of literature and proposal for patient's evaluation. Clin Ophthalmol.

[21] Danis RP, Stephens T (1997). Phototoxic reactions caused by sodium fluorescein. Am J Ophthalmol.

